# Chasing the dragon and stumbling upon an octopus: A case of heroin-induced leukoencephalopathy and reverse takotsubo cardiomyopathy

**DOI:** 10.1016/j.amsu.2021.102797

**Published:** 2021-09-04

**Authors:** Saad Ahmad, Adrian Whiting, David Song, Jonathan Vincent Reyes, Branden Ireifej, Talal Almas, Joseph J. Lieber

**Affiliations:** a Department of Internal Medicine, Icahn School of Medicine at Mount Sinai - Elmhurst Hospital Center, NY, USA; b NYU Langone Hospital - Long Island, Department of Medicine, Mineola, NY, USA; c Royal College of Surgeons in Ireland, Dublin, Ireland

**Keywords:** Heroin, Leukoencephalopathy, Takotsubo cardiomyopathy

## Abstract

The practice of heating heroin and inhaling its vapors, commonly referred to as “chasing the dragon” has been around for decades, but only gained popularity in the United States in the 1990s. Since then, there have been many documented cases of heroin-induced leukoencephalopathy (HIL) and takotsubo cardiomyopathy (TTC). This case highlights a patient with a history of heroin inhalation who presented with multiple neurological features, including bilateral upper and lower extremity weakness, blurry vision and slurred speech. Symptoms progressively worsened over the course of multiple weeks and brain imaging was consistent with toxic leukoencephalopathy secondary to heroin inhalation. Medical course was complicated by a rare associated feature of HIL: reverse Takotsubo cardiomyopathy (rTTC). Transesophageal echocardiogram demonstrated a classic basal hypokinesis and ballooning characteristic of rTTC. The patient's symptoms were treated as currently there is no guideline directed therapy for HIL or rTTC. This case demonstrated a rare and significant complication of heroin inhalation: HIL and rTTC and described potential therapies currently being studied.

## Highlights

• Heroin-induced leukoencephalopathy (HIL) is a rare neurological complication with possible contribution to the development of reverse Takotsubo cardiomyopathy (rTTC).
• HIL is a neurologic disorder associated with inhaling heroin vapors. The pathophysiology is poorly understood at this time, but the condition appears to affect mainly white matter in the brain, differentiating from other types of leukoencephalopathies.
• The diagnosis of rTTC is based on the presence of left ventricular basal hypokinesis or akinesis, acute electrocardiogram abnormalities or elevated troponin levels in the absence of any obstructive coronary disease or myocarditis.
• While there is currently no treatment regimen for HIL, antioxidants such as vitamin A, C, E, zinc, coenzyme q10, and selenium are usually recommended. However, further studies need to be conducted to assess the efficacy of the antioxidants in these patient populations.

## 1 Introduction

“Chasing the Dragon'' is a practice that emerged in Southeast Asia in the 1920s. It refers to heating up heroin's water-insoluble free base form and inhaling its vapors [1]. This practice did not fully gain traction in the United States until the 1990s. Since then, many adverse effects have been documented in the literature, including an increasingly prevalent phenomenon called heroin-induced leukoencephalopathy (HIL). We present a case on HIL that demonstrates its broad neurological effects and possible contribution to reverse Takotsubo cardiomyopathy (rTTC).

HIL is a rare neurologic disorder associated with inhaling heroin vapors. The pathophysiology is poorly understood at this time, but the condition appears to affect mainly white matter in the brain, differentiating from other types of leukoencephalopathies [2]. Fluid accumulates within the myelin sheath causing a spongiform myelinopathy, vacuolar degeneration, and demyelination that is visible on magnetic resonance imaging (MRI) [3]. Typical symptoms usually are psychological and can sometimes be mistaken for a primary psychiatric disorder, including confusion, inattentiveness and inappropriate behaviors, psychotic symptoms, stupor, comatose-like state, and even mutism [4].

In rare instances, such as our case, HIL can lead to Takotsubo cardiomyopathy (TTC). TTC is a type of acute heart failure characterized by ballooning of the apex of the heart and caused by a sudden surge of catecholamines [5]. It is often referred to as “broken heart syndrome” since it is usually seen in cases of intense acute stress. Cocaine use has been shown to cause takotsubo cardiomyopathy through the overactivation of the sympatho-adrenergic system [5,6]. Studies have shown that the catecholamine surge secondary to cocaine damages cardiomyocytes by inducing mitochondrial damage via calcium overload and through the accumulation of free radicals [6].

Even rarer so, rTTC only occurs in roughly 2% of Takotsubo cases [7]. In rTTC, instead of the typical apical ballooning and hypokinesis, basal hypokinesis is seen. It is believed that the area of hypokinesis can vary from case to case and patient to patient due to the asymmetric distribution of adrenergic receptors in the myocardium that are primarily affected by the catecholamine surge [7]. In addition, this work has been reported in accordance with SCARE [8].

## 2 Case presentation

A 37-year-old female with an 11 year history of inhalation of heroin vapers, tobacco use (unknown pack-year history), alcohol usage not consistent with alcohol abuse and history not significant for chemotherapeutic usage, presented with two weeks of progressively worsening weakness in all extremities, blurry vision, and slowed speech. The patient required the assistance of her mother to perform all activities of daily living including urination, defecation, sanitation, ambulation, and nourishment. Patient's initial neurological exam was remarkable for restlessness, decreased visual acuity, slowed movements on coordination testing, delayed response time, and slurred speech.

Two weeks after initial presentation, the patient demonstrated extensor posturing of the upper extremities, dysautonomia, inconsolable crying and rigidity. She was started on a course of antibiotics, dantrolene and high dose antioxidants including coenzyme Q10, vitamin C and vitamin E with minimal improvement. The patient was given 0.5 mg of clonazepam to help with her emotional lability likely secondary to frontal lobe involvement. Antipyretics such as acetaminophen were given in the setting of autonomic fevers. The patient became mute, areflexic, and started to experience central fevers and muscle cramps. She was briefly intubated for concerns for aspiration, but was eventually extubated. Patient also received a percutaneous endoscopic gastrostomy tube given poor overall neurological prognosis and poor deglutition.

**Fig. 1:**
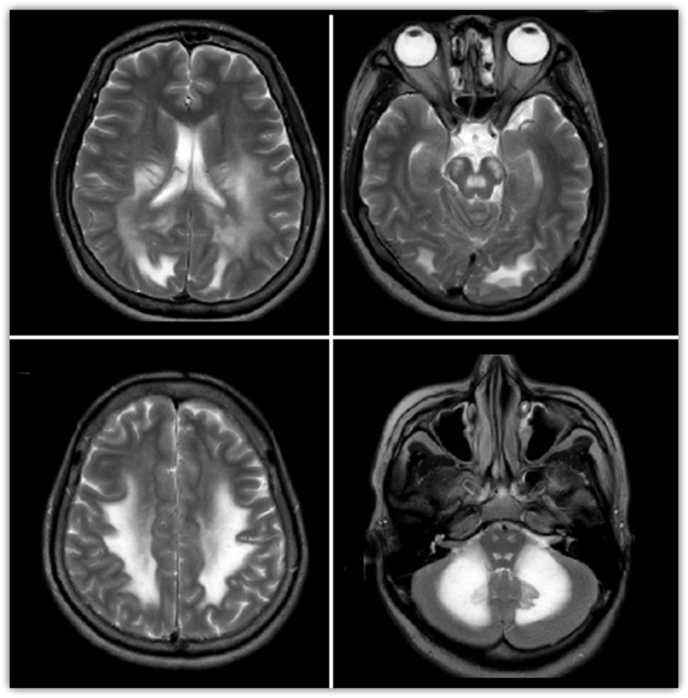
MRI of the head demonstrating extensive, symmetric T2 hyperintense signaling in the white matter of the cerebellum, brainstem, cerebral hemispheres, and some aspects of the frontal lobes.

**Fig. 2:**
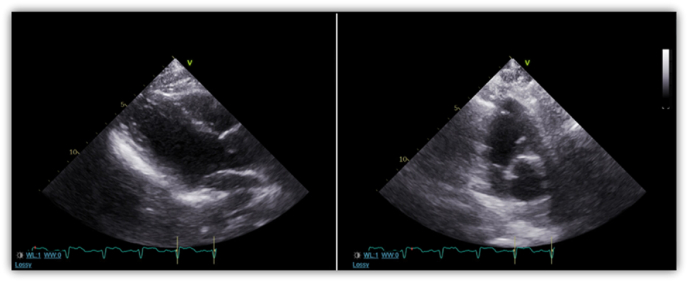
Transthoracic echocardiogram (TTE) demonstrated basal ventricular ballooning, indicating rTTC.

Lumbar puncture studies, pancultures (blood, urine, sputum, CSF), heavy metal toxicity screening, computed tomography angiography and magnetic resonance imaging (MRI) of the spine were unremarkable. Cardiac enzymes including troponin and B-type natriuretic peptide were also unremarkable. MRI of the head demonstrated extensive, symmetric T2 hyperintense signaling in the white matter of the cerebellum, brainstem, cerebral hemispheres, and some aspects of the frontal lobes (Fig. 1). Electroencephalogram showed disorganization, diffuse slowing, and sharp waves with triphasic morphology without evidence of epileptiform activity. Transthoracic echocardiogram (TTE) demonstrated basal ventricular ballooning, indicating rTTC (Fig. 2). The patient eventually demonstrated minimal improvement in speech and was clinically stable without fevers, symptomatic tachycardia, hypertension or hypotension. She was discharged to a skilled nursing facility shortly after with close outpatient monitoring with neurology and cardiology.

During the hospital course the neurology and medicine team closely monitored the patient on the general medicine floors while the patient resumed antioxidant therapy. Since discharge she has demonstrated gradual improvement in motor function and now is able to talk more consistently. The patient still requires assistance with activities of daily living.

## 3 Discussion

Since inhaling heroin vapors gained popularity in the U.S. in the 1990s, more research has been performed on HIL and TTC to elucidate their mechanisms of actions. The rationale for why inhaled heroin vapors causing leukoencephalopathy when compared to intravenous heroin is currently unknown, but it is thought to be due to a higher dose-response relationship in the vapors and the creation of toxic compounds during the heating process [9]. Some literature had variation from 1 to 30 years of heroin usage before the development of toxic leukoencephalopathy [10]. It is now known that HIL progresses through three main stages over the course of weeks to months [9]. The initial stage of HIL is primarily cerebellar, typically including pseudobulbar speech, motor restlessness, and cerebellar ataxia. The intermediate stage, occurring 2–4 weeks after onset, includes worsening of cerebellar symptoms, extrapyramidal symptoms (myoclonus and chorea), and pyramidal tract signs (hyperreflexia and spastic paresis). The terminal stage often includes akinetic mutism, central fevers, areflexia, muscle spasms and is often associated with eventual death [11]. Our patient progressed through all the distinct clinical stages. Although excessive alcohol usage and chemotherapeutics have been known to cause leukoencephalopathy, the electroencephalogram and MRI findings in our patient are pathognomonic and unique to HIL (Fig. 1, Fig. 2) [12,13].

Our patient was found to have rTTC, a condition associated with young age and neurological disease. The diagnosis of rTTC is based on the presence of left ventricular basal hypokinesis or akinesis, acute electrocardiogram abnormalities or elevated troponin levels in the absence of any obstructive coronary disease or myocarditis [14]. The underlying mechanism for rTTC is still unknown, but proposed etiologies include catecholamine-induced cardiotoxicity, as it is seen in our patient, coronary microvasculature impairment, and coronary vasospasm [14]. Recent case report from London demonstrated the role of sympathetic stimulation in the development of rTTC [15]. The patient in their case exhibited signs and symptoms of rTTC after glycopyrrolate administration, which inhibited the parasympathetic nervous system allowing the sympathetic nervous system to dominate. Thus, the patient in our case developed rTTC secondary to sympathetic hyperactivity and catecholamine surge due to heroin inhalation.

The typical presentation of rTTC is similar to acute coronary syndrome, but with no evidence of coronary obstruction [14]. Overall, patients with rTTC tend to present with less severe symptoms compared to that of TTC. Case reports have demonstrated a lower incidence of pulmonary edema, dyspnea and cardiogenic shock in rTTC when compared to TTC [13]. This observation is hypothesized due to the degree of hemodynamic changes caused by the location and the extent of the regional wall abnormality [16].

In contrast to TTC, the hallmark of rTTC is basal akinesis or hypokinesis. Our patient underwent TTE, which demonstrated basal dyskinesis suggestive of rTTC (Fig. 2). The theory behind basal hypokinesis and ballooning in rTTC is due to the higher distribution of adrenoreceptors in the basal area of the heart in younger patients. As people age and with estrogen deficiency seen in postmenopausal women, the distribution of adrenoreceptors concentrates predominantly in the apex of the heart, presenting the classic appearance of TTC in older patients [17]. The treatment for rTTC is similar to that of TTC. Most cases are treated symptomatically as TTC and rTTC tend to be transient conditions. However, in a subset of patients who experience hemodynamic instability, intra-aortic balloon pumps, cardiac stimulants, and inotropes can be deployed. Hemodynamically stable patients can be treated in a fashion similar to that of heart failure patients with the use of beta blockers, diuretics and angiotensin-converting enzyme inhibitors. The use of anticoagulation can also be considered in patients with left ventricular (LV) systolic dysfunction until LV contractility returns to normal [13].

Treatment for HIL is still unknown, but studies have shown that antioxidants may aid in the resolution of clinical and radiological findings. Despite the initial minimal improvement, we continued the course of antioxidants in hopes of improvement over time. Increased white matter lactate and response to antioxidants indicate that HIL may be related to a mitochondrial dysfunction causing demyelination and oligodendrocyte apoptosis [17]. Other recently published studies have demonstrated the use and success of antioxidants in the treatment of HIL [2]. While there is currently no treatment regimen for HIL, antioxidants such as vitamin A, C, E, zinc, coenzyme q10, and selenium are usually recommended [18]. However, further studies need to be conducted to assess the efficacy of the antioxidants in these patient populations.

## 4 Conclusion

HIL is a rare neurological complication with possible contribution to the development of rTTC that can potentially improve with antioxidant agents. Given its safety profile, we encourage clinicians to consider early initiation of antioxidant therapy for HIL. However, further studies warrant its full efficacy in the patient population.

## Funding

None.

## Ethical approval

Obtained.

## Consent

Obtained.

## Author contribution

SA, AW wrote the abstract, case, study concept, design, conclusion;

DS, JVR, BI reviewed paper, wrote discussion.

DS, TA, JL performed final edits.

## Registration of research studies

1 Name of the registry: NA
2 Unique Identifying number or registration ID: NA
3 Hyperlink to your specific registration (must be publicly accessible and will be checked): NA

## Guarantor

Talal Almas.

RCSI University of Medicine and Health Sciences.

123 St. Stephen's Green Dublin 2, Ireland.

Talalamas.almas@gmail.com.

+353834212442.

## Declaration of competing interest

None.
